# LncRNA PANDAR regulates the G1/S transition of breast cancer cells by suppressing p16^INK4A^ expression

**DOI:** 10.1038/srep22366

**Published:** 2016-03-01

**Authors:** Yi Sang, Jianjun Tang, Siwei Li, Liping Li, XiaoFeng Tang, Chun Cheng, Yanqin Luo, Xia Qian, Liang-Ming Deng, Lijuan Liu, Xiao-Bin Lv

**Affiliations:** 1 Nanchang Key Laboratory of Cancer Pathogenesis and Translational Research, Center Laboratory, the Third Affiliated Hospital, Nanchang University, Nanchang, P.R. China; 2 Department of Gastroenterology, Cancer Hospital of Jiangxi Province, Nanchang, P.R. China; 3 Department of Radiation Oncology, the Affiliated Hospital of Guilin Medical University, P.R. China; 4 Department of Medicine, GaomingHeshui Hospital, Foshan, P.R. China; 5 Department of pharmacy, Cancer Hospital of Jiangxi Province, Nanchang, P.R. China

## Abstract

It has been reported that lncRNA PANDAR (promoter of CDKN1A antisense DNA damage-activated RNA) is induced as a result of DNA damage, and it regulates the reparation of DNA damage. In this study, we investigated the role of lncRNA PANDAR in the progression of breast cancer and found that PANDAR was up-regulated in breast cancer tissues and cell lines. The knockdown of PANDAR suppresses G1/S transition of breast cancer cells. We demonstrated mechanistically that the regulation of G1/S transition by PANDAR was partly due to the transcriptional modulation of p16^INK4A^. Moreover, we showed that PANDAR impacted p16^INK4A^ expression by regulating the recruitment Bmi1 to p16^INK4A^ promoter. To our knowledge, this is the first study which showed the functional roles and mechanisms of PANDAR in regulating the progression of breast cancer. The PANDAR/Bmi1/p16^INK4A^ axis could serve as novel targets for breast cancer therapy.

Breast cancer occurs in mammary gland epithelial tissue; 99% of breast cancer occurs in women while only 1% occurs in men. It has become a threat to women’s physical and mental health^1^. The development of breast cancer is a complex multistep process associated with numerous signaling pathway alterations^2^. Accordingly, the exploration of the underlying mechanisms in breast cancer has been the subject of extensive research over past decades. However, the mechanisms of breast cancer tumorigenesis and progression are still poorly understood.

Recently, noncoding RNAs, such as microRNAs^3^,^4^,^5^,^6^,^7^,^8^ and lncRNAs^9^,^10^,^11^,^12^,^13^, have become a hotspot in the development and progress of breast cancer. However, studies on lncRNAs in breast cancer are at a preliminary stage. One of the well-known LncRNA HOTAIR is reported to be overexpressed in primary breast cancer^14^,^15^,^16^, and the expression level of HOTAIR is significantly associated with distant metastasis and poor prognosis^15^. Recently, increasing evidence have suggested that numerous lncRNAs may play critical roles in breast cancers^11^,^17^,^18^. It was reported that lncRNAs SSPRY4-IT1 and UCA1 were dysregulated in breast cancer samples and increased the proliferation of breast cancer cells^19^,^20^. Another study revealed that lncRNA EFNA3 was induced by hypoxia and that it promoted metastatic dissemination of breast cancer^21^. In addition, it was reported that lncRNA INXS induced apoptosis of breast cancer cells^22^. Although lncRNAs may have an impact on breast cancer, their detailed role and molecular mechanisms are still largely unknown.

LncRNA PANDAR was first reported by Hung *et al.*^23^. The PANDAR was located approximately 5 kilobases upstream of the CDKN1A transcription start site and was induced upon DNA damage. During DNA damage, PANDAR is induced by p53 and the knockdown of PANDAR increases the DNA damage-induced apoptosis. Recently, PANDAR was reported to control the entry and exit into and out of senescence. In proliferating cells, PANDAR and SAFA recruit PRC complexes to repress the transcription of senescence-promoting genes. Conversely, the loss of SAFA–PANDAR–PRC interactions allows the expression of the senescence program^24^. More recently, Han *et al.* reported that PANDAR was down-regulated in non-small cell lung cancer (NSCLC) and that a low PANDAR level predicted a poor prognosis^25^. However, Peng *et al.* found that PANDAR was up-regulated in hepatocellular carcinoma and that a low PANDAR level predicted a good prognosis^26^. These reports indicate that PANDAR plays complicated roles in cancers. In this study, we found that PANDAR was up-regulated in breast cancer tissues and cell lines. The knockdown of PANDAR reduced cell growth and colony formation of breast cancer cells. Mechanistically, the silence of PANDAR led to the G1/S arrest but did not affect the apoptosis of breast cancer cells. Furthermore, our results indicated that p16^INK4A^ was the downstream target of PANDAR and was responsible for PANDAR-mediated G1/S arrest. More importantly, we revealed that PANDAR enhanced the binding of Bim1 complex to p16^INK4A^ promoter and suppressed p16^INK4A^ expression. Our findings suggest that PANDAR could function as a tumor-promoting gene and regulate the cell cycle of breast cancer cells.

## Results

### PANDAR is up-regulated in breast cancer clinical samples as well as cell lines

**Figure 1:**
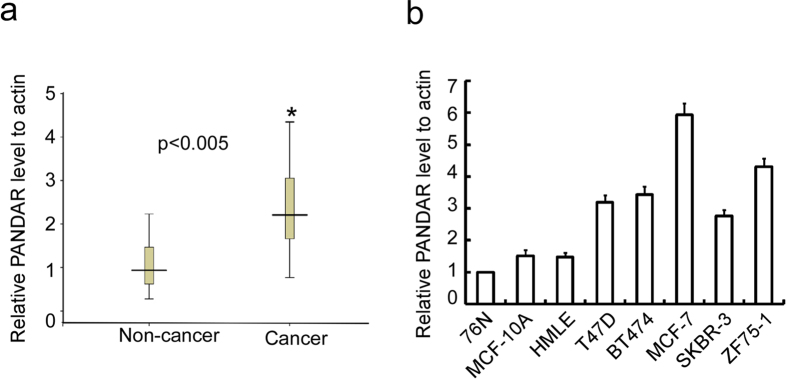
PANDAR was dysregulated in breast cancer. (**a**) PANDAR level was evaluated in twenty-four pairs of freshly frozen primary breast cancer tissues and breast cysts tissues using qRT-PCR. (**b**) PANDAR level was evaluated in breast cancer cells and immortalized epithelial cells. The results are expressed as the mean ± SD; *n* = 3, **p < 0.01.

To explore the potential role of PANDAR in breast cancer progression, we compared the PANDAR level in breast cancer tissues and non-cancerous tissues. PANDAR levels in 24 pairs of freshly frozen primary breast cancer tissues and breast cysts tissues were evaluated using qRT-PCR. As shown in Fig. 1a, PANDAR was significantly up-regulated in breast cancer compared to breast cysts tissues. We then detected the PANDAR level in a panel of breast cancer and immortalized breast cell lines. Consistent with the observation in tissues, PANDAR level was up-regulated in breast cancer cells compared with immortalized breast cells (Fig. 1b). These results indicate that PANDAR was dysregulated in breast cancer.

### PANDAR regulates the proliferation and colony formation of breast cancer cells

**Figure 2:**
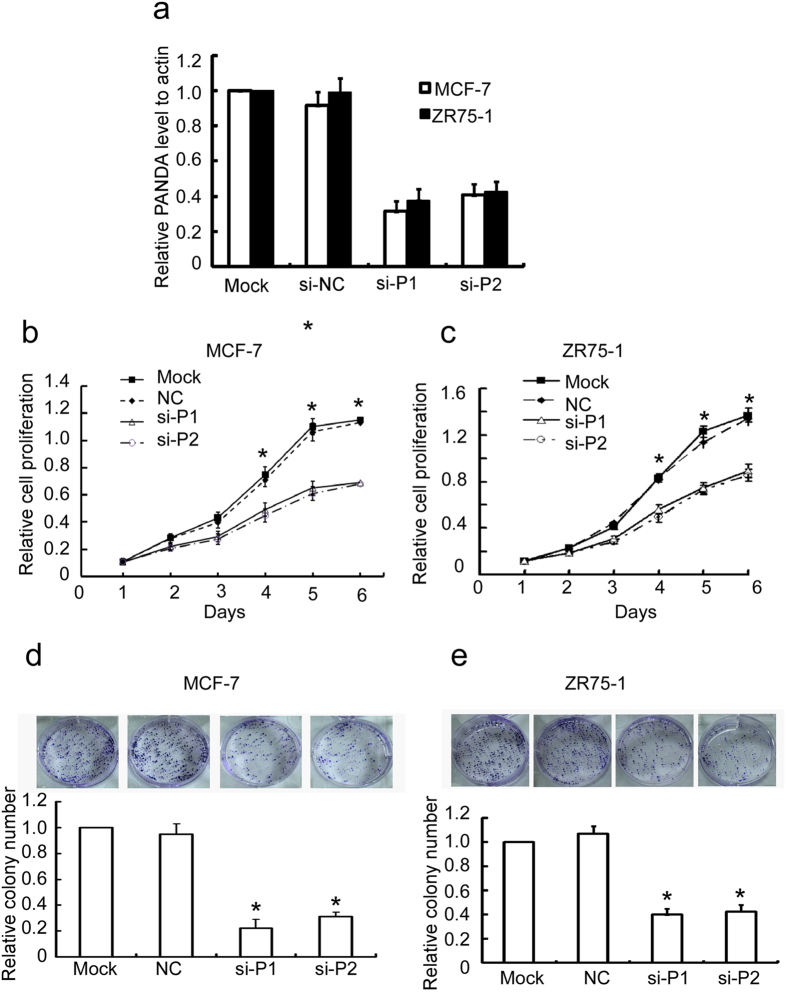
Silence of PANDAR (si-P1 or si-P2) suppresses the proliferation and colony formation of MCF-7 and ZR75-1 breast cancer cells. (**a**) MCF-7 and ZR75-1 cell lines stably silencing PANDAR were constructed and PANDAR level were evaluated with qRT-PCR. (**b**,**c**) The proliferation of MCF-7 and ZR75-1 cells stably silencing PANDAR were tested by MTT assay. (**d,e**) silence of PANDAR represses the colony formation of MCF-7 and ZR75-1 cells. The results are expressed as the mean ± SD; *n* = 3, *p < 0.05.

The above results prompted us to investigate the functional role of PANDAR in breast cancer cells. PANDAR was efficiently silenced using siRNAs (Fig. 2a) and the cell proliferation was evaluated by MTT assay. Notably, we observed a significantly reduced cell growth of MCF-7 upon PANDAR knockdown compared with the control (Fig. 2b). Accordingly, similar results were also observed in T47D cells (Fig. 2c). Furthermore, consistent with the proliferation assay, the silence of PANDAR remarkably suppressed the colony formation of both MCF-7 (Fig. 2d) and T47D cells (Fig. 2e). Altogether, these results indicate that PANDAR modulates the proliferation of breast cancer cells.

### The knockdown of PANDAR results in the G1 cell-cycle arrest of breast cancer cells

**Figure 3:**
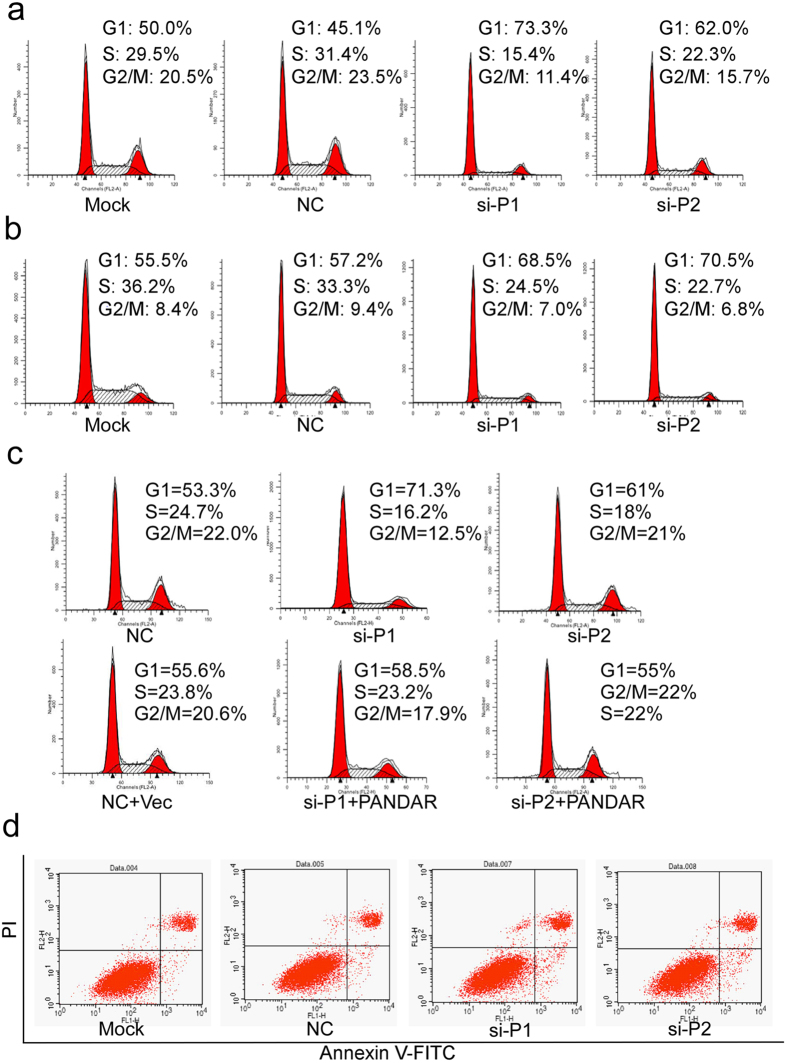
Knockdown of PANDAR resulted in the G0/G1 cell cycle arrest of breast cancer cells. MCF-7 (**a**) and ZR75-1 cells (**b**) were transiently transfected with PANDAR, GFP (NC) siRNAs or mock for 48 h and the cell cycle profiles were examined by flow cytometry. (**c**) Reintroduction of PANDAR expressing plasmid rescues PANDAR silencing exerted G0/G1 phase arrest in MCF-7 cells. (**d**) PANDAR silencing doesn’t affect the apoptosis of MCF-7 cells.

Given that silencing PANDAR suppressed the cell growth of breast cancer cells, we sought to determine its underlying mechanisms. First, we established whether the silencing PANDAR affected the cell cycle of breast cancer cells. As shown in Fig. 3a, PANDAR siRNAs transfection led to a notably elevated G1 phase ration in MCF7 cells compared to mock control, whereas negative control (NC) transfection did not exert any influence on MCF-7 cells. Moreover, this alteration of cell cycle profile by PANDAR siRNAs was almost completely rescued by the co-transfection of siRNAs resistant PANDAR plasmid in MCF-7 cells, which excluded the possible off-targeting effect by PANDAR siRNAs (Fig. 3b). The knockdown of PANDAR consistently increased the G1 phase ration in ZR-75-1 cells (Fig. 3c). Next, we examined whether PANDAR regulated the apoptosis of breast cancer cells. The silencing of PANDAR has no obvious effect on the apoptosis of both MCF-7 (Fig. 3d) and T47D cells (Data not shown). Taken together, these results indicate that PANDAR regulates the cell cycle progression but not the apoptosis of breast cancer cells.

### PANDAR regulates the G1/S transition of breast cancer cells

**Figure 4:**
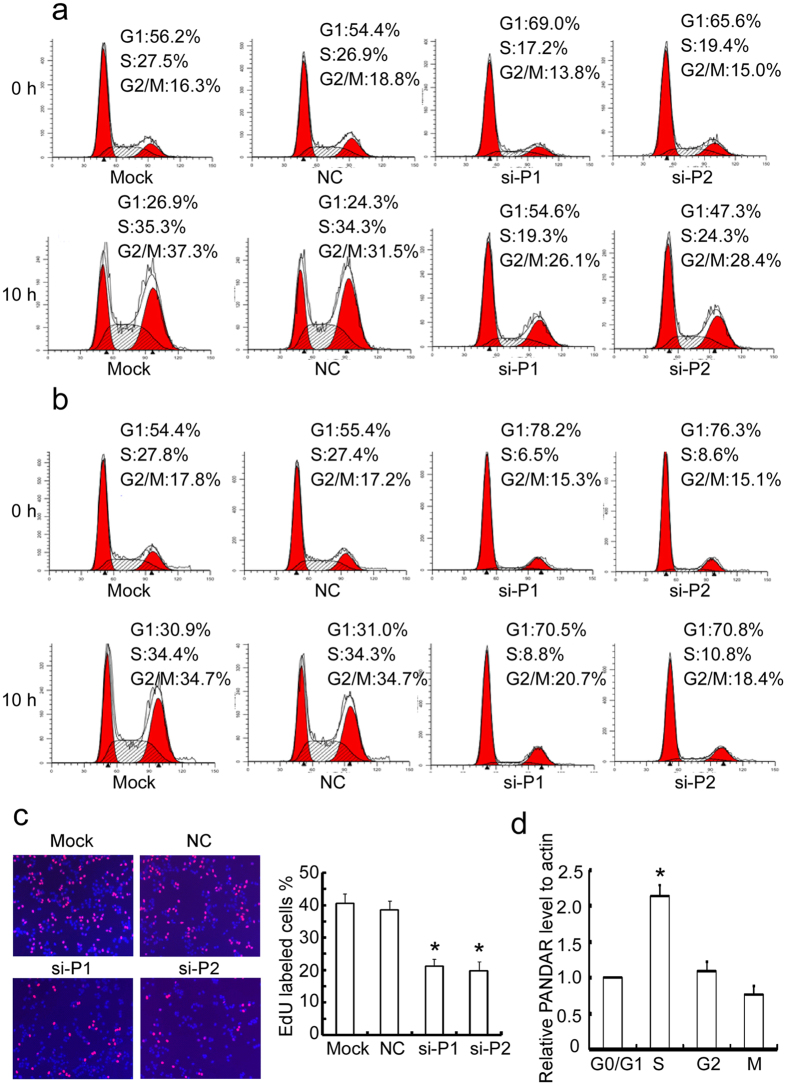
Silence of PANDAR suppresses the G1/S transition of breast cancer cells. MCF-7 (**a**) and ZR75-1 cells (**b**) transiently transfected with PANDAR, GFP (NC) siRNAs or mock for 48 h were treated with or without 100 ng/mL of nocodazol for 10 h and the cell cycle profiles were examined by flow cytometry. (**c**) MCF-7 cells transiently transfected with PANDAR, GFP (NC) siRNAs or mock for 48 h were incubate with EdU for 2 h and the labeled cells were counted under microscope. (**d**) MCF-7 cells were synchronized at G1, S, G2 and M phase by double thymidine treatment and the PANDAR level at these phases was evaluated with qRT-PCR. The results are expressed as the mean ± SD; *n* = 3, *p < 0.05.

We determined whether the increased G1-phase cells by PANDAR knockdown was due to the arrested G1/S transition or the increased mitotic exit. The percentage of cells in the G1 phase was measured by flow cytometry using MCF7 and ZR75-1 cells treated with nocodazole, which arrests cells at M phase. As shown in Fig. 4a,b, the percentage of cells in the G1 phase was remarkably increased by PANDAR knockdown in MCF-7 (Fig. 4a) and ZR75-1 cells (Fig. 4b), indicating that the reduced G1 phase cells entered S phase upon PANDAR knockdown. The silencing of PANDAR did not affect the mitotic exit of MCF-7 cells (data not shown). In addition, by using EdU dye assay, we observed a notable reduction of cells entering the S phase upon silencing PANDAR compared to negative control (Fig. 4c). The expression of cell cycle regulators often orchestrates cell cycle progression^27^. Hence, we examined the expression of PANDAR in MCF7 cells synchronized in G0/G1, S, G2 and M phases respectively. PANDAR expression level increased from G1 to S phase and remained steady or changed slightly during G2, M, and G1 phases, implying that PANDAR plays an important role in the G1/S transition of breast cancer cells. Altogether, these results indicate that the down-regulation of PANDAR represses the G1/S transition of breast cancer cells.

### PANDAR regulates the G1/S transition of breast cancer cells through p16^INK4A^

**Figure 5:**
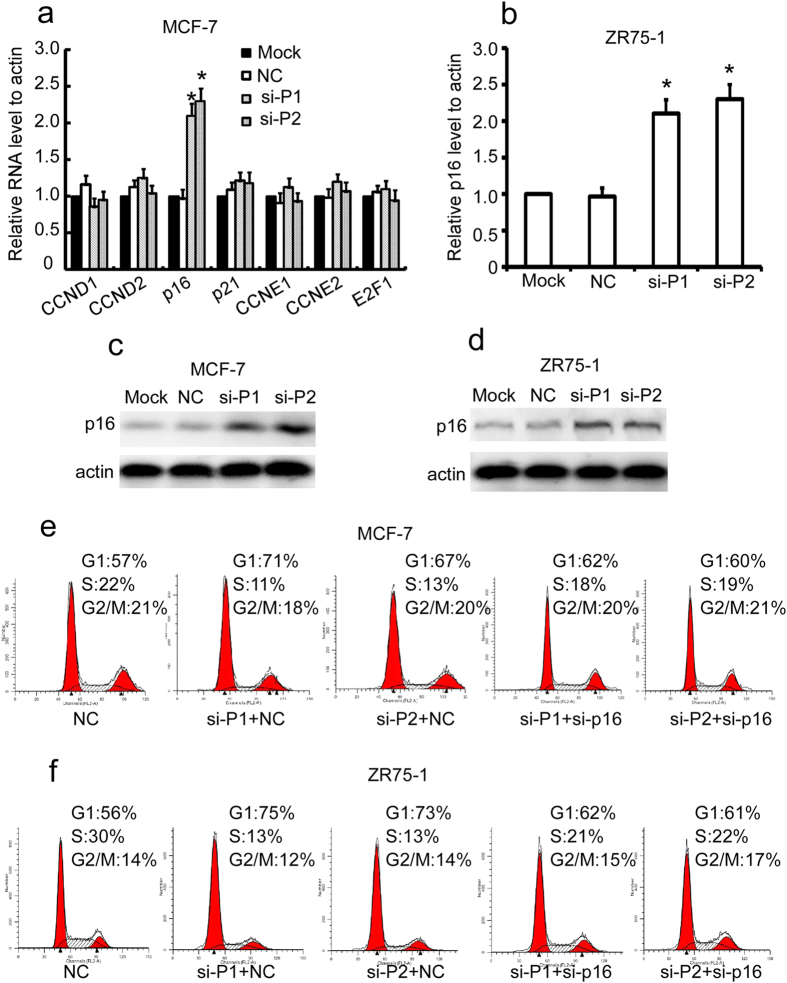
PANDAR regulates the G1/S transition of breast cancer cells partly through the upregulation of p16^INK4A^ expression. (**a**) MCF-7 cells were transiently transfected with si-PANDAR, si-GFP (NC) or mock for 48 h and the mRNA level of indicated genes were evaluated by qRT-PCR. (**b**) ZR75-1 cells were transiently transfected with PANDAR, GFP (NC) siRNAs or mock for 48 h and the mRNA level of p16^INK4A^ was evaluated by qRT-PCR. (**c**,**d**) MCF-7 and ZR75-1 cells were transiently transfected with PANDAR, GFP (NC) siRNAs or mock for 48 h and the protein level of p16^INK4A^ was evaluated by western blot. All the gels were run under the same conditions. The corresponding full-length blots were included in supplementary figures 1 and 2. (**e**,**f**) MCF-7 and ZR75-1 cells were transiently transfected with indicated siRNAs for 48 h and the cell cycle profiles were tested by flow cytometry. The results are expressed as the mean ± SD; *n* = 3, *p < 0.05.

To explore the potential signaling pathway regulated by PANDAR, we screened a panel of G1 phase-related regulators by qRT-PCR. We found that silencing PANDAR remarkably increased the expression of p16 in MCF-7 cells (Fig. 5a). This observation was also confirmed in ZR75-1 cells (Fig. 5b). Consistently, the protein level of p16^INK4A^ was also obviously elevated upon silencing PANDAR in MCF-7 (Fig. 5c) and ZR75-1 cells (Fig. 5d). More importantly, the increase of G1 phase ration by silencing PANDAR was reversed in part by the co-transfection of p16^INK4A^ into MCF-7 (Fig. 5e) and ZR75-1 cells (Fig. 5f). Taken together, these results suggest that PANDAR may control G1/S transition by regulating p16^INK4A^ expression.

### PANDAR regulates p16^INK4A^ expression via modulating the binding of Bmi1 to its promoter

**Figure 6:**
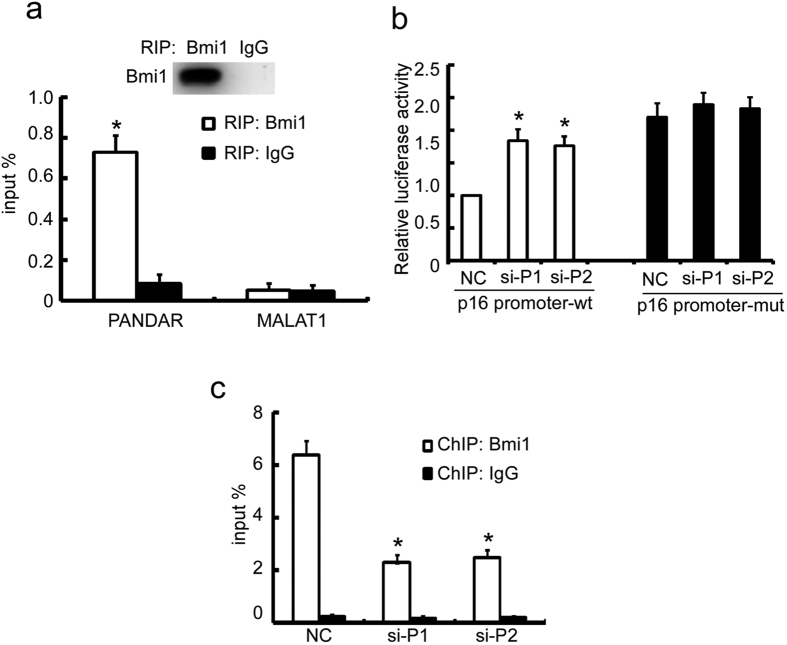
PANDAR controls p16^INK4A^ expression through regulating the recruitment of Bmi1 to its promoter. (**a**) MCF-7 cells were lysed and the clarified lyses were subjected to RIP with indicated antibodies. The enrichment of indicated lncRNAs was evaluated with qRT-PCR. (**b**) MCF-7 cells stably silencing PANDAR were transfected with p16^INK4A^ promoter (p16 promoter-wt) or its mutant deleting Bmi1 response element (p16 promoter-mut) for 24 h and the luciferase activity were evaluated. (**c**) MCF-7 cells were transiently transfected with indicated siRNAs for 48 h. Endogenous ChIP was performed with BMI1 or isotopic IgG and the enrichment of bound p16^INK4A^ pro moter was evaluated with qRT-PCR. The results are expressed as the mean ± SD; *n* = 3, **p < 0.01, *p < 0.05.

It was reported that PANDAR interacts and impacts Bmi1-PRC1 complex activity and regulates the expression of a panel of its targeting genes^24^. In addition, Bmi1 was reported to directly repress the transcription of p16^INK4A ^28. Therefore, we reasoned that PANDAR impacted p16^INK4A^ expression by regulating the recruitment Bmi1 to p16^INK4A^ promoter. First, we examined the interaction of PANDAR and Bmi1 using Bmi1. MALAT1, a well-known lncRNA was used as a negative control. As shown in Fig. 6a, PANDAR was enriched by Bmi1 antibody but not by IgG, whereas MALTA1 wasn’t be enriched by Bmi1. We then investigated whether PANDAR impacted the promoter activity of p16^INK4A^. A−1.2-kb genomic sequence upstream of the p16^INK4A^ ATG initiation codon was inserted into a promoterless luciferase reporter vector, pGL3-basic. This plasmid was co-transfected with pRL control plasmid and siPANDAR/NC into MCF-7 cells and the relative luciferase activity was examined. Indeed, the knockdown of PANDAR remarkably up-regulated the luciferase activity compared to NC (Fig. 6b). To further confirm that PANDAR regulates p16^INK4A^ expression through Bmi1, we determined whether PANDAR knockdown affected the binding of Bmi1 to p16^INK4A^ promoter in MCF-7 cells using chromatin immunoprecipitation (ChIP). In agreement with previous reports^28^, Bmi1 antibody strongly enriched the p16^INK4A^ promoter compared to IgG. Upon the silencing of PANDAR, the enrichment of Bim1 to p16^INK4A^ was obviously reduced (Fig. 6c). Altogether, these results suggest that PANDAR suppresses p16^INK4A^ expression by facilitating the binding of Bmi1 to p16^INK4A^ promoter.

## Discussion

It is clear that <2% of the total genome sequence are protein-coding genes, and at least 98% of the genome are transcribed into non-coding RNAs (ncRNAs)^29^. The functional roles of most of these transcripts, especially the lncRNAs, remain obscure. In particular, the involvement of lncRNAs in breast cancer pathogenesis and progression has not been widely studied.

In our study, we found that lncRNA PANDAR was significantly up-regulated in breast cancer tissues as well as breast cancer cells. Our findings indicate that PANDAR may function as a cell cycle regulator of breast cancer cells. The silencing of PANDAR suppresses the G1/S transition of breast cancer cells, leading to decreased cell growth.

Because PANDAR level was notably elevated in breast cancer tissues compared to non-cancerous tissues, we inferred that PANDAR might play important roles in tumor biology. First, we investigated the expression of PANDAR in a panel of breast cancer cell lines and immortalized normal breast epithelial cells. We found that PANDAR was up-regulated in breast cancer cells compared to normal breast epithelial cells, agreeing with our findings in breast cancer tissues. We then examined the functional contribution of PANDAR to tumor-like characteristics such as proliferation and apoptosis. Indeed, the silencing of PANDAR suppressed cell proliferation of MCF-7 and ZR75-1 cells. We demonstrated mechanistically that PANDAR regulates the G1/S transition of breast cancer cells. The knockdown of PANDAR results in a significant G0/G1 cell cycle arrest. Han *et al.* reported that PANDAR was down-regulated, and it played a tumor-suppressing role in NSCLC^25^. The contradictory functions of PANDAR in cancers as observed in our results and Han’s report might be due to the differences between the properties of breast cancer and NSCLC. Moreover, it was reported that the expression of PANDAR was induced in fibroblast upon gene stress, and the silencing of PANDAR attenuated DNA damage induced apoptosis^23^. In our results, the silencing of PANDAR did not affect the apoptosis of breast cancer cells, suggesting that appropriate gene stress is required for PANDAR silencing-mediated apoptosis of breast cancer cells.

The molecular mechanisms of lncRNAs are diverse. They exert their functions mostly by interacting with proteins or binding with miRNAs as sponges. PANDAR was found to interact with NF-YA, SAFA and Bmi1, and function as a transcriptional co-suppressor^24^. Hence, we screened a panel of G1/S transition-related regulators and found that p16^INK4A^ was regulated at transcriptional level in MCF-7 cells. In addition, we found that p16 could partially rescue PANDAR silencing mediated G1/S arrest, indicating that p16^INK4A^ plays an important role in PANDAR-exerted regulation of cell cycle.

Bmi1, the first functionally identified PcG member, was originally identified as an oncogene cooperating with c-Myc in a murine lymphomagenesis model^30^,^31^. In humans, Bmi1 plays different roles in cell cycle, cell immortalization and senescence^32^. Numerous studies demonstrate that Bmi1 was up-regulated in a variety of cancers and has a positive correlation with clinical stage/grade and poor prognosis^33^,^34^. Bmi1 controls cell cycle by regulating p16^INK4A^ and some other cell cycle regulators^32^. p16^IINK4A^ inhibits binding of cyclin D to CDK4/6 resulting in the suppression of RB activity and cell cycle arrest^35^. Recently, it was reported that PANDAR control Bmi1 activity by regulating PRC-Bmi1 interaction^24^. In this study, we found that the silencing of PANDAR could suppress p16^INK4A^ expression at transcriptional level. Moreover, the co-transfection of si-PANDAR and si-p16 could partially abrogate si-PANDAR-induced G1 phase arrest. ChIP assay results indicate that the silencing of PANDAR could cause an attenuation of BMI1 binding at the promoter of p16. These results indicate that p16^INK4A^ is a bona fide target of PANDAR/Bmi1-regulated gene. Our findings provide a potential novel mechanism through which PANDAR boosts tumor cell proliferation, partly due to the up-regulation of p16^INK4A^ through Bmi1.

Our study suggests that PANDAR could function as a tumor-promoting gene in breast cancer by regulating G1/S transition. Furthermore, we demonstrated that PANDAR-mediated promotion of cell growth is at least partially through the regulation of p16^INK4A^. Our findings may supply a strategy for targeting the PANDAR/Bmi1/p16^INK4A^ axis as a novel therapeutic application for breast cancer patients.

## Materials and Methods

### Cell Culture and Sample Collection

Human breast cancer cell lines (BT474, SK-BR3, MCF-7, T47D and MDA-MB-231) were cultured in Dulbecco’s modified Eagle’s medium (DMEM, Life) supplemented with 10% fetal bovine serum (BI). Immortalized breast epithelial cell lines (MCF-10A, 76n and HMLE) were cultured in DMEM/F-12 1:1 mix supplemented with human insulin (10 μg/ml), epidermal growth factor (20 ng/ml), cholera toxin (50 μl), hydrocortisone (0.5 μg/ml), and horse serum (5%).

All cell lines were incubated in a humidified chamber with 5% CO_2_ at 37 °C. Twenty-four fresh primary breast cancer tissues and non-cancerous tissues were obtained at the time of diagnosis before any therapy from Cancer Hospital of Jiangxi Province (Nanchang, China). The clinical processes were approved by the Ethics Committee of Nanchang University, and informed consent was collected from each patient.

### RNA extraction and quantitative real-time PCR (qRT-PCR)

qRT-PCR was performed as described previously^36^. Total RNA of tissue specimens was isolated using Trizol^TM^ (Life Technology, USA) according to the manufacturer’s instructions. First-strand cDNA was synthesized using MLV transcriptase Kit (Life Technology, USA). qRT-PCR was performed using gene specific primers (Supplementary Table 1) for indicated genes with SYBR^®^ Premix Ex Taq™ II (Takara, Japan). The PCR condition was as follows: 95 °C for 4 min, followed by 45 cycles of 95 °C for 20 s, 60 °C for 20 s and 70 °C for 30 s.

### Oligonucleotide transfection

Small interfering RNAs (siRNAs) specifically targeting human PANDAR were designed according to previously validated oligonucleotides^23^ and were synthesized by GenePharma (Shanghai, China). The p16 siRNA was purchased from Qiagen (USA). siRNA against GFP (si-GFP) bought from GenePharma (Shanghai, China) were used as the negative control (NC). The siRNAs were transfected into cells at a working concentration of 50 nmol/L using the RNAiMAX reagent (Life Technology, USA) according to the manufacturer’s instructions.

### Western blotting

The above procedures were performed as previously described^37^. The gels were run under the same experimental conditions and the original whole gel blots were included in the supplementary data.

### Cell cycle profile assay

Cell cycle profile assay was performed as previously described^38^. Briefly, cells were harvested by trypsinization and collected by centrifugation. Cells were washed twice with phosphate-buffered saline (PBS) and fixed in 1 ml of 70% ethanol at 4 °C for 1 h or overnight. Cells were washed twice with PBS/1% bovine serum albumin (BSA) and then incubated with 1 ml of PBS/1% BSA containing 30 mg/ml propidium iodide and 0.25 mg/ml RNase A for 30 min at room temperature. Cells were analyzed for DNA content by flow cytometry using a cytomics FACSCalibur (BD Biosciences, USA). The data were analyzed using Modifit.

### G1/S transition assay

As previously stated^39^, cells transfected for 48 h with control siRNAs or PANDAR siRNAs were added to 100 ng/ml of nocodazole (Sigma-Aldrich, St Louis, MO, USA) for 12 h. Cells were harvested by trypsinization and fixed with 70% ethanol at 4 °C for 1 h to overnight. Cell cycle distributions were measured by staining the cells with propidium iodide (PI), followed by analysis on a flow cytometer (BD Biosciences, USA).

### EdU dying

EdU dying was performed using Cell-Light™ EdU Apollo^®^567 *In Vitro* Imaging Kit (Ribobio Technology, China). Cells transfected with siRNAs for 48 h were added to 50 μM EdU for 2 h. The cells were then washed twice with PBS and fixed with 4% paraformaldehyde. Cells with EdU inserted into the chromosome were labeled with Apollo567 according to the manufacturer’s instructions. The labeled cells were counted under a microscope.

### Vector constructions

The 1.2 K *p16* promoter that spanned from nucleotide +1 to nucleotide −1214 upstream of the starting codon ATG^28^ was amplified from MCF-7 cells and cloned into pGL3-Basic plasmi. PANDAR was amplified from MCF-7 cells and cloned into pcDNA3.1 plasmid. The p16 promoter deleting Bmi1 response element (pGL3-p16-dBRE) and siRNA resistant PANDAR were obtained using a Quik-Change Site-Directed Mutagenesis Kit (Stratagene) according to the manufacturer’s instructions.

### Luciferase report assay

This procedure was previously described but with slight modification^40^. Briefly, vectors delivering p16 promoter and the control vector pRL-TK (Promega) coding for Renilla luciferase were cotransfected with PANDAR siRNAs or negative control into MCF-7 cells for 48 h. The luciferase activity was measured 48 h later using the Dual-Luciferase Reporter Assay System (Promega). The firefly luciferase values were normalized to Renilla, and the ratios of firefly/Renilla values were presented. The experiments were performed independently in triplicate.

### ChIP assay

ChIP assay was performed as previously described^41^. Briefly, MCF-7 cells were crosslinked in 1% formaldehyde solution for 10 min at room temperature, followed by the addition of 125 mM of glycine for 5 min. ChIP assay was performed with ChIP assay kit (26156, Thermo) according to the manufacturer’s instructions. The nucleoprotein complexes were digested to yield DNA fragments ranging from 200 to 500 bp using the Micrococcal Nuclease in the kit. Two micrograms of normal IgG were used as the negative control while anti-Bmi1 was used for each immunoprecipitation. The immunoprecipitates were eluted and reverse crosslinked, after which the DNA fragments were purified. Immunoprecipitated and input DNAs were subjected to qRT–PCR analysis. The primers used for amplifying the Bmi1 recognizing site were previously validated^28^ and are as follows: forward - 5-CCCATTTTCCTATCTGC-3; reverse - 5-CTAGTTCAAAGGATTCC-3.

### RNA immunoprecipitation (RIP)

Cells were lysed in NP-40 lysis buffer (50 mM Tris-HCl, ph 7.4, 150 mM NaCl, 1% NP-40 and Protease inhibitor cocktail and RNAse inhibitor) and cleared lysates were immunoprecipitated with indicated anti-Bmi1 and IgG antibodies. Immune complexes were purified with Protein-A/G-coupled dynabeads (Life Technologies). Immunoprecipitated and input RNA were isolated by Trizol^TM^ LS reagent. RNA was reverse transcribed by MLV transcriptase Kit (Life Technologies). cDNA was used as a template in qRT–PCR amplifications with PANDAR specific primers.

### Study approval

The use of human lung cancer tissues was reviewed and approved by the Ethical Committee of Cancer Hospital of Jiangxi Province and was performed in accordance with the approved guidelines. The evaluation of the PANDAR level of the cancer tissues using qRT-PCR was in accordance with the approved guidelines. Informed consents were obtained from the patients.

### Statistical analysis

All statistical analyses were performed using SPSS for Windows, version 16.0 (SPSS). All values from the *in vitro* assays are expressed as the mean ± SD of at least three independent experiments or replicates. P values were calculated using the two-tailed Student’s test. A p value <0.05 is considered statistically significant.

## Additional Information

**How to cite this article**: Sang, Y. *et al.* LncRNA PANDAR regulates the G1/S transition of breast cancer cells by suppressing p16^INK4A^ expression. *Sci. Rep.* **6**, 22366; doi: 10.1038/srep22366 (2016).

## Supplementary Material

Supplementary Information
