# Supplementary material for: LncRNA PANDAR regulates the G1/S transition of breast cancer cells by suppressing p16INK4A expression
Source: Sci Rep. 2016 Mar 1;6:22366. doi: 10.1038/srep22366 (PMC4772134; doi:10.1038/srep22366)
Supplement: Supplementary Information [file srep22366-s1.pdf]

LncRNA PANDAR regulates the G1/S transition of breast cancer cells by suppressing p16^{INK4A} expression

Yi Sang[1,#,] Jianjun Tang[2,#], Siwei Li[5,#] Liping Li[1], XiaoFeng Tang[1], Chun Cheng[1], Yanqin Luo[1], Xia Qian[1], Liang-Ming Deng[4], Lijuan Liu[3], Xiao-Bin Lv[1,*]

1 Nanchang Key Laboratory of Cancer Pathogenesis and Translational Research, Center Laboratory, the Third Affiliated Hospital, Nanchang University, Nanchang, P.R. China.

2 Department of Gastroenterology, Cancer Hospital of Jiangxi Province, Nanchang, P.R. China.

3 Department of pharmacy, Cancer Hospital of Jiangxi Province, Nanchang, P.R. China.

4 Department of Medicine, GaomingHeshui Hospital, Foshan, P.R. China

5 Department of Radiation Oncology, the Affiliated Hospital of Guilin Medical University, P.R. China

# These authors contribute equally to this work

* Correspondence to: Xiao-Bin Lv, nclvxiaobin@sina.cn

Supplementary Table 1. qRT-PCR primers list.

Name	Sequence
PANDAR Forward	TGCACACATTTAACCCGAAG
PANDAR Reverse	CCCCAAAGCTACATCTATGACA
CCND1 Forward	GCTCCTGGTGAACAAGCTCAA
CCND1Reverse	TTGGAGAGGAAGTGTTCAATGAAA
CCND2 Forward	TGATGGGGAAGGAGTACAAA
CCND2 Reverse	TTGGAAGAAGAAGCAGGATG
p16 Forward	GTGGGTCCCAGTCTGCAGTTAAG
p16 Reverse	CATCATCATGACCTGGTCTTCTAGG
p21 Forward	GCAGACCAGCATGACAGATTTC
p21 Reverse	GGATTAGGGCTTCCTCTTGGA
CCNE1 Forward	CTTCACAGGGAGACCTTTTAC
CCNE1 Reverse	CATTCAGCCAGGACACAATAG
CCNE2 Forward	TGATGGTGCTTGCAGTGAAGAGGA
CCNE2 Reverse	CACAAGGCAGCAGCAGTCAGTATT
E2F1 Forward	ATGTTTTCCTGTGCCCTGAG
E2F1 Reverse	ATCTGTGGTGAGGGATGAGG
MALAT1 Forward	GACGGAGGTTGAGATGAAGC
MALAT1 Reverse	ATTCGGGGCTCTGTAGTCCT
Actin Forward	TGAAGTGTGACGTGGACATC
Actin Reverse	GGAGGAGCAATGATCTTGAT

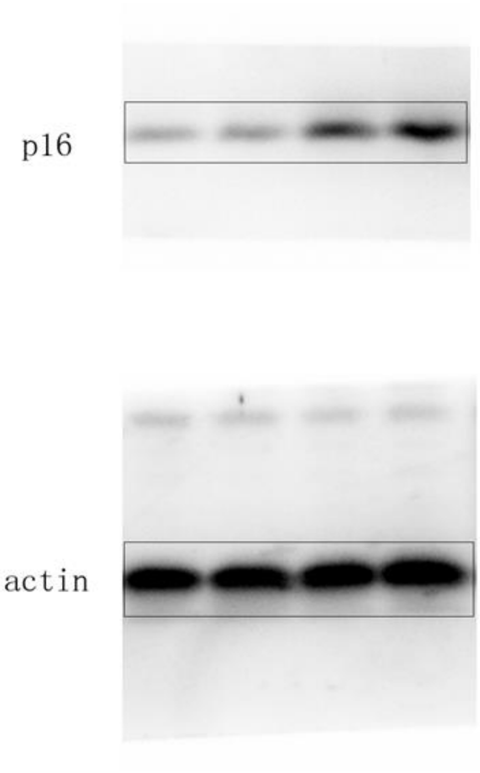

Supplementary Figure 1.

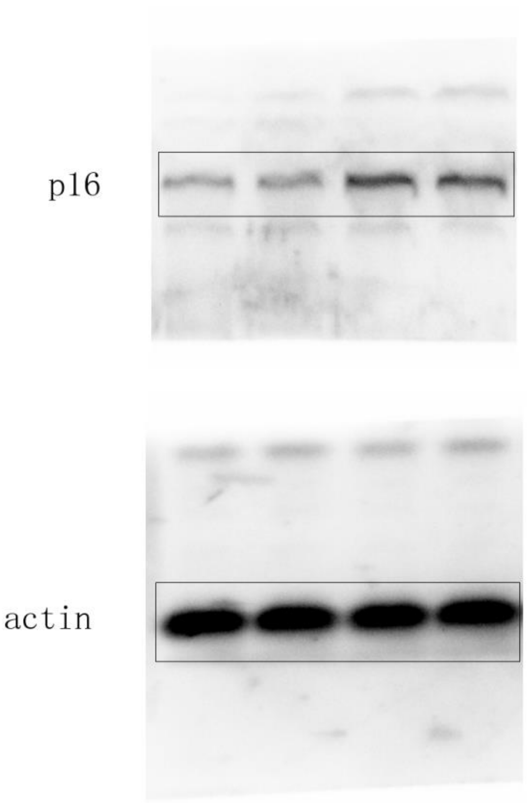

Supplementary Figure 2.
